# Chitosan, polyethylene oxide/polycaprolactone electrospun core/shell nanofibrous mat containing rosuvastatin as a novel drug delivery system for enhancing human mesenchymal stem cell osteogenesis

**DOI:** 10.3389/fmolb.2023.1220357

**Published:** 2023-07-13

**Authors:** Fariba Ghasemvand, Mahboubeh Kabiri, Vahideh Hassan-Zadeh, Abdolreza Simchi

**Affiliations:** ^1^ Department of Cell and Molecular Biology, Kish International Campus, University of Tehran, Kish, Iran; ^2^ Department of Biotechnology, College of Science, University of Tehran, Tehran, Iran; ^3^ Department of Cell and Molecular Biology, Faculty of Biology, College of Science, University of Tehran, Tehran, Iran; ^4^ Department of Materials Science and Engineering, Sharif University of Technology, Tehran, Iran

**Keywords:** nanofiber, core/shell structure, coaxial electrospinning, drug release, bone tissue engineering

## Abstract

**Introduction:** Due to the potential positive effects of rosuvastatin (RSV) on human mesenchymal stem cells (MSCs) osteogenesis and new bone regeneration, it is crucial to develop a suitable carrier that can effectively control the release profile of RSV. The primary objective of this study was to introduce a novel drug delivery system based on core/shell nanofibrous structures, enabling a sustained release of RSV.

**Methods:** To achieve this, coaxial electrospinning was employed to fabricate chitosan (CS)+polyethylene oxide (PEO)/polycaprolactone (PCL) nanofibrous mats, wherein RSV was incorporated within the core of nanofibers. By optimizing the relevant parameters of the electrospinning process, the mats’ surface was further modified using plasma treatment. The fibers’ shape, structure, and thermal stability were characterized. The wettability, and degradation properties of the fabricated mats were also examined. *In vitro* studies were conducted to examine the release behavior of RSV. Additionally, the capability of MSCs to survive and differentiate into osteocytes when cultured on nanofibers containing RSV was evaluated.

**Results:** Results demonstrated the successful fabrication of CS + PEO + RSV/PCL core/shell mats with a core diameter of approximately 370 nm and a shell thickness of around 70 nm under optimized conditions. Plasma treatment was found to enhance the wettability and drug-release behavior of the mats. The nanofibrous structure, serving as a carrier for RSV, exhibited increased proliferation of MSCs and enhanced osteogenic differentiation.

**Conclusion:** Therefore, it can be concluded that CS + PEO + RSV/PCL core/shell nanofibrous structure can be utilized as a sustained-release platform for RSV over an extended period, making it a promising candidate for guided bone regeneration.

## 1 Introduction

Bone is a dense, living tissue that protects internal organs, helps the body support its weight, produces blood cells, and stores minerals. Bone has the ability to regenerate and heal itself under normal physiological conditions. However, bone’s natural self-healing capacity is not sufficient in extreme cases, such as accidents, injuries, and bone diseases, such as osteoporosis. In recent decades, there has been a surge in interest in bone tissue engineering (BTE) methods as potential alternatives to conventional medical treatments for bone disorders and defects. Scientists have focused on creating an optimal framework for skeletal tissue, serving as a temporary structure to support cell adhesion, proliferation, and differentiation (Srouji et al., 2011; Shalumon et al., 2015; Kalani et al., 2019; Nikolova and Chavali, 2019; Abbasi et al., 2020; Collins et al., 2021).

Because of their similarity to natural extracellular matrix (ECM) morphology, high surface area, and enhanced cellular response, the development of biopolymer nanofibers, from biomaterials such as chitosan (CS), polyethylene oxide (PEO), polycaprolactone (PCL), and collagen have gained significant interest for bone regeneration (Pakravan et al., 2012; Rubert et al., 2014; Roozbahani et al., 2015; Jalaja et al., 2016). Due to the low inflammatory response, good mechanical strength, and enhanced cell reactivity, CS fibers have attracted much interest in BTE scaffolding (Surucu and Sasmazel, 2016; Ma et al., 2019). However, the limited osteogenic capacity of CS fibers poses a significant challenge for bone repair applications. To improve the interaction between the cell matrix and the cells, a variety of osteogenic agents have been incorporated into nanofibrous scaffolds (Kim et al., 2014; Kang et al., 2015; Lai et al., 2018). Recently, there has been an increased interest in the use of statins and other lipid-lowering medications to promote the formation of new bones (Shepherd et al., 2003; Pişkin et al., 2009; Farsaei et al., 2012).

Rosuvastatin (RSV, Mw = 485.1 Da) is recognized as one of the most potent hydrophilic statins available, known for its effectiveness in reducing cholesterol levels. In comparison to other commonly used statins such as simvastatin and atorvastatin, RSV has been associated with fewer side effects and can be used over a longer period of time. (Shepherd et al., 2003; Pişkin et al., 2009; Laurencin et al., 2014; Kalani et al., 2019; Yousefi et al., 2022). It has been observed that an appropriate dose of RSV can have a positive impact on osteogenesis and new bone production (Kalani et al., 2019; Rezazadeh et al., 2019). RSV induces osteoblast differentiation by interfering in the BMP-2/SMAD signaling pathway, leading to enhanced BMP-2 gene expression and ALP activity. It also delays osteoblast apoptosis (Laurencin et al., 2014). However, higher doses may be cytotoxic by reducing the formation of cholesterol, a molecule essential for the integrity of cell membranes. High concentrations can also cause an inflammatory response and slow the bone healing process (Monjo et al., 2010a; Monjo et al., 2010b; Rezazadeh et al., 2019). Therefore, the design of suitable platforms to control the release profile of RSV seems inevitable to promote osteogenic differentiation (Laurencin et al., 2014; Bharathi et al., 2022). To decrease the initial drug release and regulate the dosage of the released drug, core/shell nanofibers containing the drug in the core can be utilized (Sill and Von Recum, 2008; Vacanti et al., 2012; Lee et al., 2016; Boroojen et al., 2019; Ghazalian et al., 2022; Farhaj et al., 2023). In recent years, coaxial electrospinning has gained importance for the production of nanofibers with core/shell structures. Coaxial electrospinning, as opposed to single-nozzle electrospinning, can provide various benefits in drug delivery applications, including enhancing drug delivery effectiveness and safeguarding pharmaceuticals in harsh conditions (Buzgo et al., 2013; Perez and Kim, 2015; Cheng et al., 2018; Heydari et al., 2018; Zahedi et al., 2019; Zandi et al., 2020). For example, Yousefi et al. (Yousefi et al., 2022) constructed a PCL/CS core/shell nanofibrous mat as a drug delivery system (DDS) for RSV, with the CS layer serving as the shell and containing 5 wt% of RSV. While the addition of CS had increased the hydrophilicity of the DDS leading to increased bioavailability of RSV, their result showed that the addition of CS in the shell had decreased the mechanical properties of the mat. In addition, CS in the shell had caused a burst release of the drug in the acidic pH range. Almost after 24 h, their core/shell DDS reached a plateau phase in drug release. Due to the CS shell’s pH-responsive characteristic, they proposed DDS as a beneficial approach in drug delivery technology. A core/shell nanofiber was also created by Kalani et al. (Kalani et al., 2019) as a carrier for controlling RSV release and osteogenic differentiation *in vitro*. They used silk fibroin (SF) as the core and polyvinyl alcohol (PVA) as the shell for their structures. According to the release profile, hydrophilic RSV encapsulation in the SF layer contributed significantly to lowering the initial burst release and subsequently improving its sustainability throughout the 15-day experiment.

In this study, we have presented a novel DDS capable of sustained release of RSV over an extended period, which has not been previously reported. To achieve this, a coaxial electrospinning technique was employed to fabricate CS + PEO/PCL nanofibrous mats with a core/shell structure. CS and PEO were blended with RSV in the preparation of the core due to their ease of electrospinning and miscibility with RSV (Pakravan et al., 2012; Barzegari and Shariatinia, 2018; Sohi et al., 2018). Additionally, to modify the predominantly PCL-covered surface of the fabricated mats, plasma treatment was employed (Sharifi Ferdoey et al., 2014; Recek et al., 2016). The size, shape, structure, and thermal stability of the fibers were evaluated using several characterization techniques. Furthermore, human mesenchymal stem cells (MSCs) were cultivated on the resulting RSV-containing nanofibers, and the vitality and osteogenic differentiation of these cells were assessed.

## 2 Materials and methods

### 2.1 Materials

PCL (Mw = 80,000 g/mol), CS (Mw = 50,000 g/mol), PEO (Mw = 40,000 g/mol), chloroform (CF), dimethylformamide (DMF), and acetic acid (99.5%) were supplied by Merck (Germany). The RSV drug was purchased from Pursina Company (Iran).

### 2.2 Fabrication of CS + PEO/PCL core/shell nanofibers containing RSV

To prepare the core solution, CS (70 mg) and PEO (30 mg) were dissolved in 2.5 mL of aqueous acetic acid and 2.5 mL of distilled water and stirred at room temperature for 2 h. Then, RSV (5 mg) was added to the CS + PEO solution and stirred for 60 min. To prepare the shell solution, PCL (10 w/v) was dissolved in CF and DMF with a volume ratio of 9:1.

To produce core/shell nanofibers, the solutions were fed into a coaxial stainless-steel spinneret consisting of two concentrically arranged needles. The flow rates of the core and shell solutions were set to 0.5 mL/h. The high-voltage generator applied a voltage of 18 kV, and the distance between the coaxial spinneret and the aluminum collection foil was 12 cm. To produce bead-free and highly uniform nanofibers of CS + PEO/PCL, some key electrospinning variables, including the proportions of the components in each solution, applied voltage, the distance between the tip and the collector, and the flow rates, were optimized by experimenting (Table 1). The PCL, CS + PEO nanofibers, and CS + PEO/PCL core/shell nanofibers without RSV were also prepared for comparison purposes. To improve the surface hydrophilicity, the electrospun nanofibers were treated with air plasma using an electronic plasma cleaner (Diener, Germany). The nanofibers were placed in the chamber of the plasma cleaner, and plasma discharge was performed for 40 s at a high-frequency power of 40 kHz at an air pressure of 0.7 mbar. The samples’ description and their assigned codes are summarized in Table 2.

**TABLE 1:** Electrospinning conditions to fabricate CS + PEO/PCL core/shell nanofibers containing RSV.

Run^a^	Voltage (kV)	Distance (cm)	Flow rate^b^ (mL/h)	Fiber morphology	Fiber diameter (nm)
1	16	10	0.5	No fiber formation	-
2	16	11	0.5	No fiber formation	-
3	16	12	0.5	Thick fibers	741 ± 15
4	18	10	0.5	Thick fibers	654 ± 12
5	18	11	0.5	Non-uniform fibers	496 ± 47
6^c^	18	12	0.5	Uniform fibers	370 ± 12
7	20	10	0.5	Beads formation	291 ± 14
8	20	11	0.5	Beads formation	207 ± 9
9	20	12	0.5	Beads formation	187 ± 12

^a^ Shell solution: CS, and PEO, with a weight ratio of 70:30 in aqueous acetic acid and distilled water (1:1) containing 5% RSV, with respect to the weight of CS + PEO, compounds. Shell solution: 10% (w/v) PCL, in CF, and DMF (9:1).

^b^ Flow rates of both core and shell solutions were considered the same.

^c^ Optimal processing conditions.

**TABLE 2:** The code and the corresponding description of each fibrous mat fabricated in this study.

Sample name	Description of fabricated fibrous mat^a^
M1	CS + PEO
M2	CS + PEO + RSV
M3	PCL
M4	CS + PEO/PCL
M5	CS + PEO + RSV/PCL
M6	Plasma-treated CS + PEO + RSV/PCL

^a^ CS, chitosan; PEO, polyethylene oxide; RSV, rosuvastatin; PCL, polycaprolactone; +, blended; and/: core/shell morphology.

### 2.3 Characterizations

Field emission scanning electron microscope (SEM, VEGA, TESCAN, Czech Republic) and transmission electron microscope (TEM, CM30, Philips, Netherlands) were used to examine the morphology/structure of the fabricated fibers. A Fourier transform infrared spectroscope (FTIR, Tensor 27, Bruker, Germany) was used to determine the presence of various compounds in the structure of the fabricated nanofibers. Thermogravimetric analysis (TGA) was performed using a thermal analyzer (PerkinElmer, United States) under an argon atmosphere at 50°C–600°C with a heating rate of 10°C/min.

To measure wettability, the water contact angle of the fabricated mats was determined using the sessile drop method. To quantify the water uptake of the mats, a portion of each sample (W_o_) was immersed in PBS (20 mL, pH = 7.4) and kept at 37°C for 24 h. Afterward, the filter paper was used to remove the samples’ surface water, which was then immediately weighed (W_t_). The following equation was used to calculate the amount of water uptake that each sample absorbed:

$$Water\;uptake\;\left(\%\right)=\frac{W_{t}-W_{0}}{W_{0}}\times 100$$ (1)

Also, each sample (W_o_) was dipped in PBS at 37°C to measure the *in vitro* degradation. Samples were taken out of PBS at each interval, fully dried, and weighed (W_d_). The following equation was used to determine weight loss as a percentage:

$$Weight\;loss\;\left(\%\right)=\frac{W_{0}-W_{d}}{W_{d}}\times 100$$ (2)

### 2.4 Drug entrapment efficiency and drug release profile

To establish a calibration curve, a dilution series ranging from 5 to 50 μg/mL of pure RSV was prepared. The UV absorption of each solution was measured using a UV-visible spectrophotometer (Lambda 850, PerkinElmer, United States) at 242 nm. A calibration curve was then depicted using the linear regression method.

To determine the drug encapsulation effectiveness, 10 mg of the CS + PEO + RSV/PCL core/shell mat was fully dissolved in 5 mL of aqueous acetic acid. The UV-visible spectrophotometer was used to obtain the solution’s absorbance value at 242 nm. Using the calibration curve, the drug content that was loaded during the electrospinning process was determined. Afterward, the CS + PEO + RSV/PCL core/shell mats were used to investigate the kinetics of drug release. For this purpose, 5 mg of the mat was transferred into an Eppendorf (Amicon Ultra-4 Centrifugal Filter Unit, Mw cutoff 10 kDa) containing 4 mL of PBS and kept at 37°C. Drug release was performed for 90 days. At certain times, the PBS solution was entirely refreshed. The UV-visible spectrophotometer was used to measure the absorbance value of each solution at 242 nm, and the drug content was determined using the calibration curve.

The mechanism of RSV release from the fabricated mat was also studied using the Korsmeyer-Peppas model (Dash et al., 2010). In this model, the fraction of drug released (*M*_*t*_ */M*_*∞*_) is expressed as a power function of time (*t*) as follows:

$$\frac{M_{t}}{M_{\infty }}=kt^{n}$$ (3)

where *M*_*t*_ and *M*_*∞*_ are the cumulative mass of the drug released at time *t* after definite time, respectively. *k* and *n* are the release constant and exponent, respectively.

### 2.5 Culture of MSCs

Dulbecco’s modified Eagle’s medium (DMEM, Gibco-Invitrogen, 2D cell culture) was used to cultivate human MSCs obtained from the Stem Cell Technology Research Center (Tehran, Iran). This medium was supplemented with 10% fetal bovine serum (FBS) and 1% penicillin/streptomycin/amphotericin-B (Invitrogen Corp, United States). Up until confluence, cells were kept in a humidified CO_2_ incubator at 37°C and given fresh media every 3 days. In this work, passage 3 cells were utilized.

### 2.6 Cell morphology, viability, and proliferation assays

For cell viability analysis, MSCs (5 × 10^3^ cells/cm^2^) were seeded on a 24-well tissue culture plate (TCP) as control and on different fabricated mats. Cell viability was assessed at 1, 3, and 5 days using the MTT test. After specific time points, the cells were rinsed with PBS and then the medium was replaced with 10% MTT reagent in high glucose DMEM for 3 h. Afterward, the medium was removed, and the formed formazan crystals were washed with DMSO and transferred into 96-well plates. A UV-visible spectrophotometer (Cary 100, Australia) was used to detect their absorbance at 570 nm. Each sample was examined three times.

The spreading of cultured MSCs (10 × 10^3^ cells/cm^2^) and their morphology following incubation on the samples were also investigated. To do this, the scaffolds that had been seeded with cells for 3 days were rinsed twice with PBS, fixed for 45 min in 2.5% glutaraldehyde, and then exposed to various concentrations of ethanol (50, 60, 70, 80, and 96%) for 20 min. The dehydrated scaffolds were then sputtered with gold and examined by SEM.

### 2.7 Cell differentiation

On each scaffold, MSCs were seeded at a concentration of 2×10^4^ cells/cm^2^. After 24 h of incubation, the culture medium was changed to an osteogenic culture medium (3D cell culture) that contained dexamethasone (0.1 M), ascorbic-2-phosphate (0.2 mM), glycerophosphate, and high glucose DMEM. The culture media was replaced every 2 days for 21 days.

#### 2.7.1 Evaluation of osteogenic gene expression

The expression of genes related to osteogenesis was examined using real-time reverse transcriptase-polymerase chain reaction (RT-PCR) according to the protocol proposed by Kalani et al. (Kalani et al., 2019). Following 7, 14, and 21 days of culture in an osteogenic medium, total RNA was extracted from MSCs-seeded samples using the Trizol reagent. The obtained RNA was then completely mixed with DEPC water after being precipitated with isopropanol. By measuring absorbance at 260 and 280 nm, the RNA concentration and purity of each sample were determined. Complementary DNA (cDNA) was synthesized from 1 to 5 μg RNA in a 20-μL reaction from each sample according to the instructions of the manufacturer of the BON mRNA first Strand cDNA Synthesis kit (Stem Cell Technology Research Center, Tehran, Iran). After dilution of cDNA in distilled water, RT-PCR analysis was performed using BON- High-Specificity qPCR master mix (Stem Cell Technology Research Center, Tehran, Iran) according to the manufacturer’s instructions in the stepOne Plus from Thermo Fisher Scientific analyzer (United States). Table 3 shows the detailed primer sequences for the important genes used in this work. Each reaction was carried out three times, and the resulting data were quantified using the 2^−ΔΔCt^ technique. The relative expression of each gene was normalized using the endogenous housekeeping gene GAPDH.

**TABLE 3:** Primer pair sequences for real-time RT-PCR.

Gene	Sequence
RUNX2-F	GCCTTCAAGGTGGTAGC
RUNX2-R	CGTTACCCGCCATGACA
H-ALP-F	GCACCTGCCTTACTAAC
H-ALP-R	AGACACCCATCCCAT C
Osteonectin-F	AGGTATCTGTGGGAGCT
Osteonectin-R	ATTGCTGCACACCTTC
Osteopontin-F	GCC​GAG​GTG​ATA​GTC​TGG​TT
Osteopontin-R	TGAGGTGATGTCCTCGTC TG
H-GAPDH-F	CTCTCTGCTCCTCCTGTT
H-GAPDH-R	ACGACCAAATCCGTTGAC

#### 2.7.2 Qualification and quantification of calcium deposition

Using Alizarin red staining (ARS), the quantity of calcium precipitated on the various scaffolds was calculated. The seeded MSCs (2 ×10^4^ cells/cm^2^) on each mat after cultivation for 7, 14, and 21 days in the osteogenic medium were fixed with cold absolute methanol for 5 min and rinsed with distilled water. The calcium deposited on the different substrates was determined in a dark environment by adding 200 µL of ARS solution to each sample and being incubated on a shaker for 30 min. Following a thorough distilled water wash, the samples were dried for 5 min at 30°C before being examined under an inverted microscope (Omax LED, United States). Each stained sample was transferred to a fresh 24-well plate and subjected to 0.5 mL of acetic acid solution (10%) on an orbital shaker for 30 min in order to measure ARS. Using an EL-Reader (PowerWave XS2, Bio Tek, United States), the absorbance was determined at 405 nm after the full dissolution of the attached dye.

Osteogenic differentiation and mineralization were also measured by a calcium content assay kit (Pars Azmoun, ALP kit, Iran). As explained above, the seeded and cultivated MSCs at different times were washed with cold PBS twice. Samples were transferred to vials and 150 μL hydrochloric acid (0.6 N) was added. Samples were shaken for 60 min in a dark environment. Vials were centrifuged for 15 min at 14,000 rpm. The supernatants were used in the calcium content assay kit test and the optical density (OD) was measured at 570 nm using the EL-Reader.

### 2.8 Quantification of the ALP activity

The intracellular ALP activity of MSCs was measured using an ALP kit (Pars Azmoun ALP kit, Iran). The same aforementioned procedure was employed to seed and cultivate MSCs (2 ×10^4^ cells/cm^2^) on each sample. After twice washing with cold PBS, the samples were placed in vials, and 150 μL cold RIPA solution was added. Samples were shaken for 60 min in a dark environment. Then vials were centrifuged for 15 min at 14,000 rpm. The supernatants were used in the ALP kit test and the OD was measured at 405 nm using the EL-Reader.

### 2.9 Statistical analysis

The mean and standard deviation (SD) of each set of data were reported. GraphPad Prism software was used to perform two-way ANOVA for multiple comparisons. The statistical significance was defined as *p* < 0.05.

## 3 Results and discussion

**FIGURE 1:**
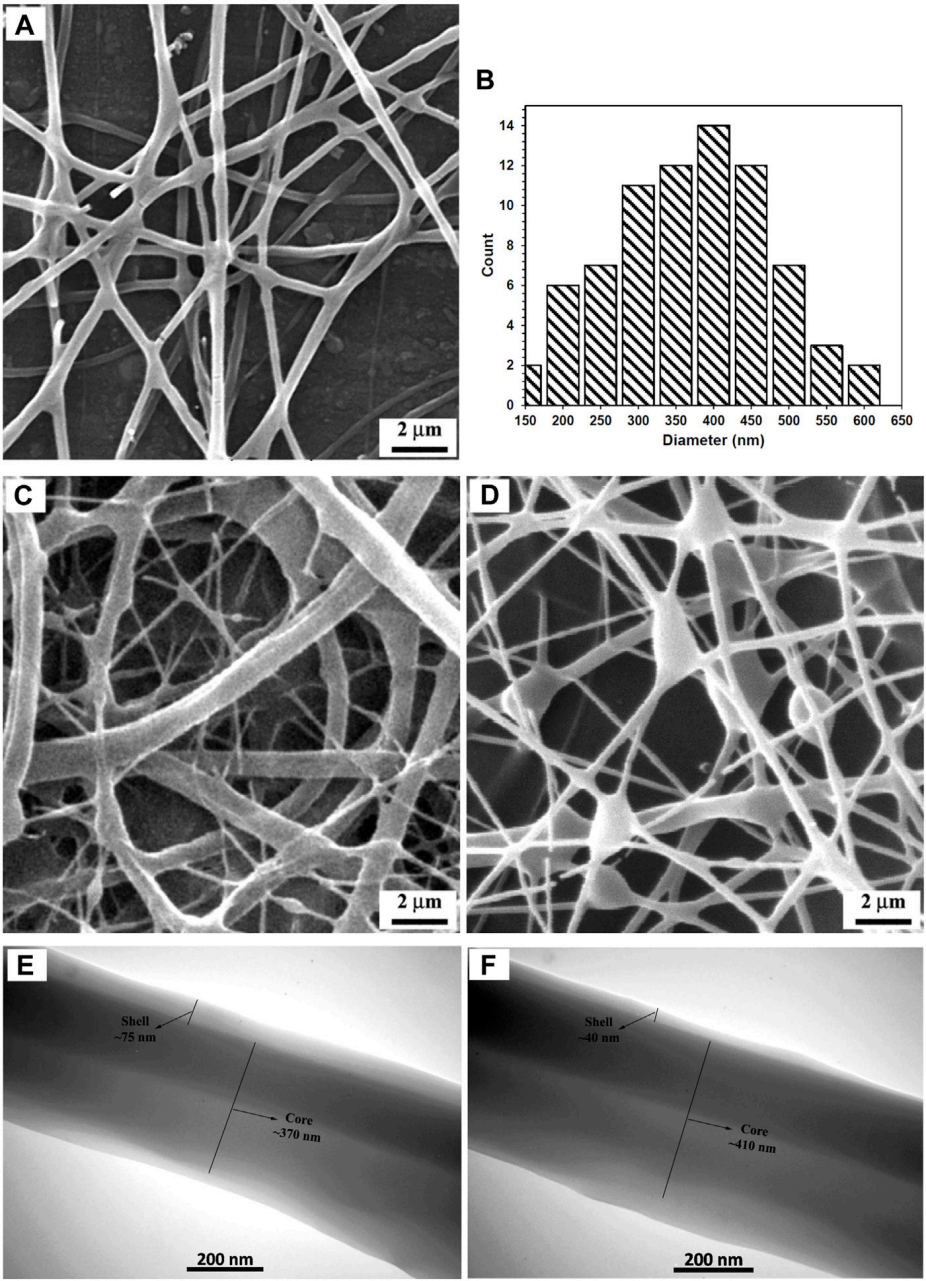
**(A, B)** SEM micrograph and corresponding fiber diameter distribution of the fabricated CS + PEO + RSV/PCL nanofibers under optimal electrospinning parameters (Run 6), **(C, D)** SEM micrographs showing the formation of non-uniform fibers (Run 5) and beads (Run 7) under other processing conditions, **(E, F)** TEM micrographs of the fabricated CS + PEO + RSV/PCL nanofibers (Run 5). See Table 1 for descriptions of different conditions.

A representative SEM micrograph and the corresponding histogram of fiber diameter distribution of the fabricated CS + PEO + RSV/PCL mat (i.e., sample M5) after single electrospinning under optimal electrospinning parameters (Table 1) are shown in Figures 1A, B, respectively. The fibers are round, smooth, and with no beads. On average, the fibers are approximately 370 nm in size. Since there are no micro- or nanoparticles in this image, it can be concluded that the core/shell structure has been successfully formed. These results align with findings reported in other studies (Surucu and Sasmazel, 2016; Ozkan and Sasmazel, 2018; Ma et al., 2019). For example, Ma et al. (Ma et al., 2019) fabricated PCL/CS core/shell nanofibers with a stable emulsion system. They stated that the average size of the fabricated nanofibers was between 200 and 700 nm. However, it must be noted that some undesired morphologies such as the presence of non-uniform fibers and beads were found in the current study under other tested processing conditions (Figures 1C, D). TEM investigations were conducted to further validate the formation of the core/shell configuration in the nanofibers. The core/shell structure of CS + PEO + RSV/PCL nanofibers is shown in Figures 1E, F. The shell layer is approximately 70 nm thick, while the average diameter of the core is 350 nm. Ozkan et al. (Ozkan and Sasmazel, 2018) reported the same observation. They concluded that in the coaxial PCL/CS core/shell nanofibers, the core material is darker than the shell layer as inferred from TEM micrographs. This indicates that the core was entirely covered by the PCL layer and the border between core and shell materials was detected.

**FIGURE 2:**
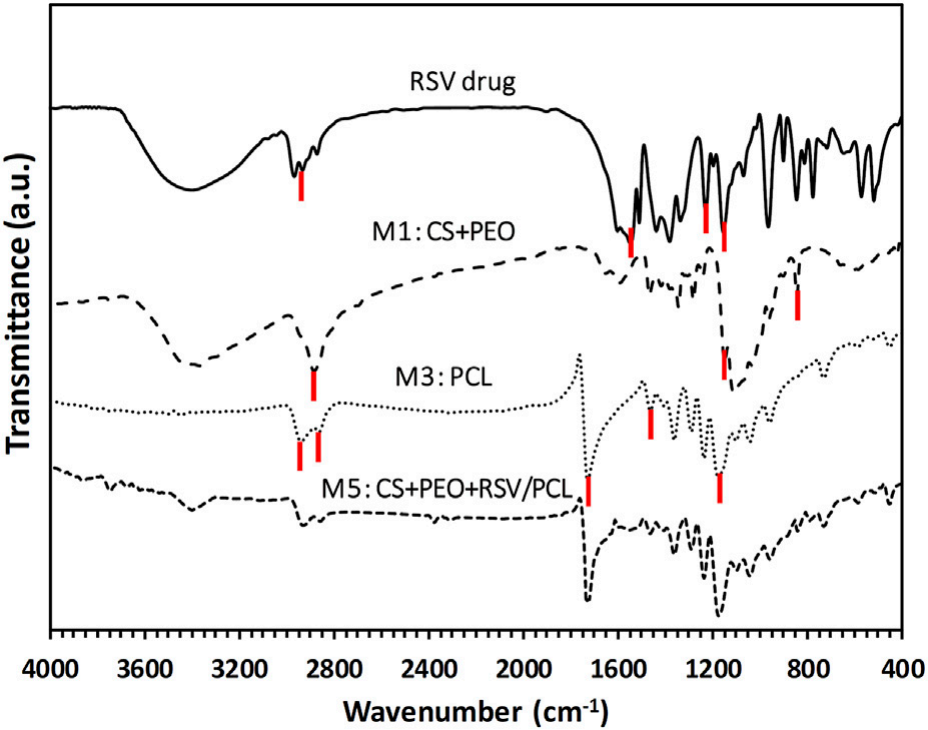
FTIR spectra of different samples.

The FTIR spectrum of pure RSV, as well as the spectra of M1 (i.e., CS + PEO), M3 (i.e., pure PCL), and M5 (i.e., CS + PEO + RSV/PCL) nanofibers, are displayed in Figure 2. In the case of pure PCL, the peaks at 2,970, and 2,860 cm^−1^ are related to the C-H stretching vibration of the hydrocarbon. The peaks at 1725, and 1,160 cm^−1^ correspond to the C=O stretching vibration and the C-O functional group of PCL. The bending absorption characteristic of the CH2-methylene group is about 1,460 cm^−1^ (Shoja et al., 2015; Zahedi et al., 2019). On the other hand, in the FTIR spectrum of M1 nanofibers, the saccharide structure of CS is represented by the peaks at 840, and 1,150 cm^−1^ (Kriegel et al., 2009). PEO shows an absorption band around 2,880 cm^−1^, which is assigned to the C-H stretching vibration (Pakravan et al., 2012). The most significant peaks in the FTIR spectrum of RSV are seen at 2930 cm^−1^ for C-H stretching, 1,540 cm^−1^ for C=C stretching, 1,228 cm^−1^ for C-F stretching, and 1,152 cm^−1^ for S=O stretching (Rezazadeh et al., 2019). Comparatively, the FTIR spectrum of M5 core/shell nanofibers contains the main peaks of the constituents, including RSV, CS, PEO, and PCL, confirming the existence of all the components in this mat.

**FIGURE 3:**
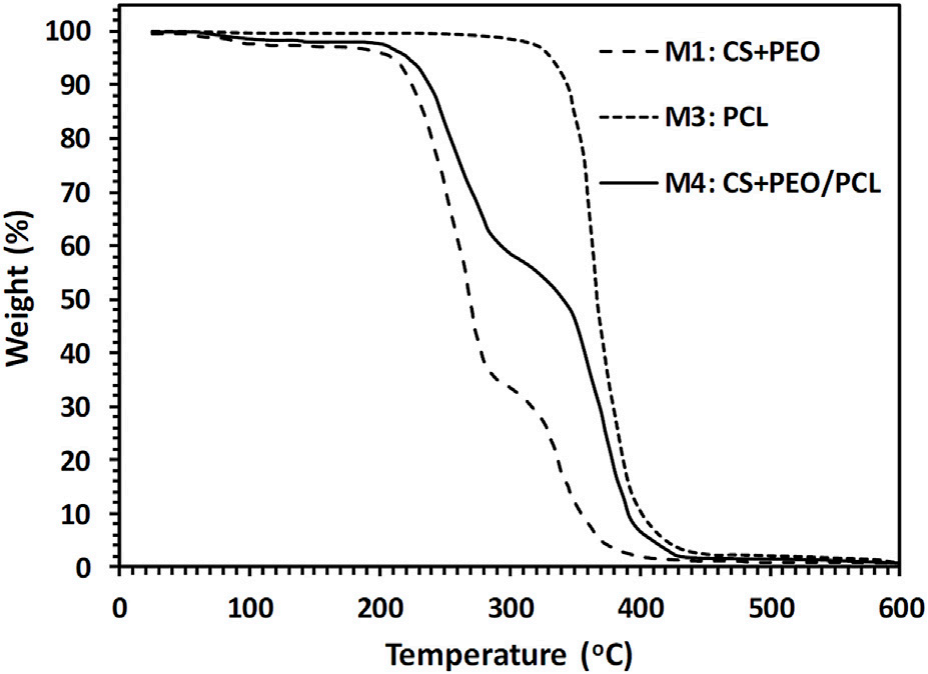
TGA curves of different mats fabricated by electrospinning.

The amount of CS, PEO, and PCL in the produced nanofibers was measured using TGA. The obtained TGA curves for the fabricated M1, M3, and M4 nanofibers mats are illustrated in Figure 3. Table 4 gives a summary of the mats’ decomposition temperatures and weight loss percentages. As seen in Figure 3, M3 nanofibers (i.e., pure PCL) exhibit a one-step weight loss of 99.4% in the temperature range of 280°C–490°C. M1 nanofibers consisting of CS and PEO compounds show a three-step weight loss. First, a 1.9% weight loss in the temperature range of 60°C–110°C, which is a consequence of water evaporation. The thermal decomposition of CS chains is responsible for the second reduction (65.3%). The third reduction (31.9%) can be attributed to the thermal decomposition of the PEO component. Figure 3 also suggests that in the CS + PEO/PCL core/shell nanofibers (sample M4), the peaks of weight reduction correspond to the decomposition of both CS + PEO and PCL. The results of TGA indicate that 43.7% CS + PEO (the second-stage weight loss peak) and 54.6% PCL (the third-stage weight loss peak) exist in the mat made of CS + PEO/PCL core/shell nanofibers. These findings are in agreement with the shell and core solution concentration ratio used to fabricate the core/shell nanofibers.

**TABLE 4:** TGA results for different mats fabricated in this study.

Sample	T_onset_-T_end_ (°C)	Weight loss (%)
M1	60–110	1.9
	180–300	65.3
	300–450	31.9
M3	280–490	99.4
M4	60–110	1.1
	180–320	43.7
	320–480	54.6

**FIGURE 4:**
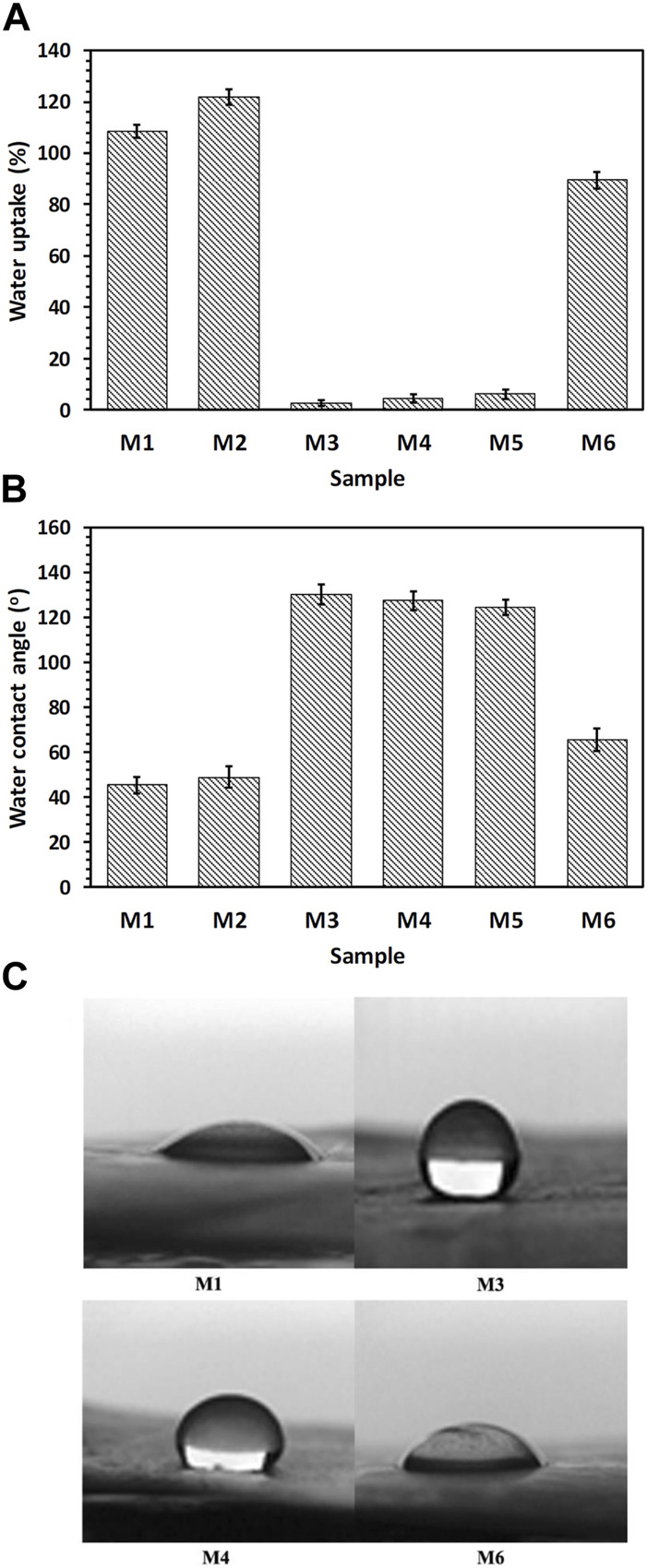
**(A)** Water uptake, **(B, C)** water contact angle measurements, showing hydrophilicity of the fabricated nanofibrous mats (see Table 1).

The results of the samples’ wettability (i.e., water absorption content and contact angle) are displayed in Figure 4. As expected water absorption was maximal for the CS + PEO nanofibers with and without RSV (samples M1, and M2) because both CS and PEO are hydrophilic (Figure 4A). However, the hydrophilicity of the nanofibers exhibited a decreasing trend for pure PCL and CS + PEO/PCL mats both with and without RSV (i.e., samples M4, and M5). This decrease can be attributed to the inherent hydrophobic nature of PCL. However, interestingly, the water uptake value exhibited a significant increase after plasma treatment of the CS + PEO + RSV/PCL nanofibers (sample M6). Similar changes in hydrophilicity can be observed in the images obtained from contact angle measurement. M1 and M2 nanofibers composed of CS and PEO with and without RSV have relatively lower water contact angles (i.e., ∼45, and ∼49^o^, respectively), while this value is higher for the M3 (∼130^o^), M4 (127^o^), and M5 core/shell nanofibers with and without RSV (∼124^o^, Figure 4B). The plasma-treated CS + PEO + RSV/PCL nanofibers (sample M6) exhibit a decreased water contact angle. According to Ref. (Chuah et al., 2015), a water contact angle between 40 and 70^o^ is ideal for cell adhesion and proliferation.

**FIGURE 5:**
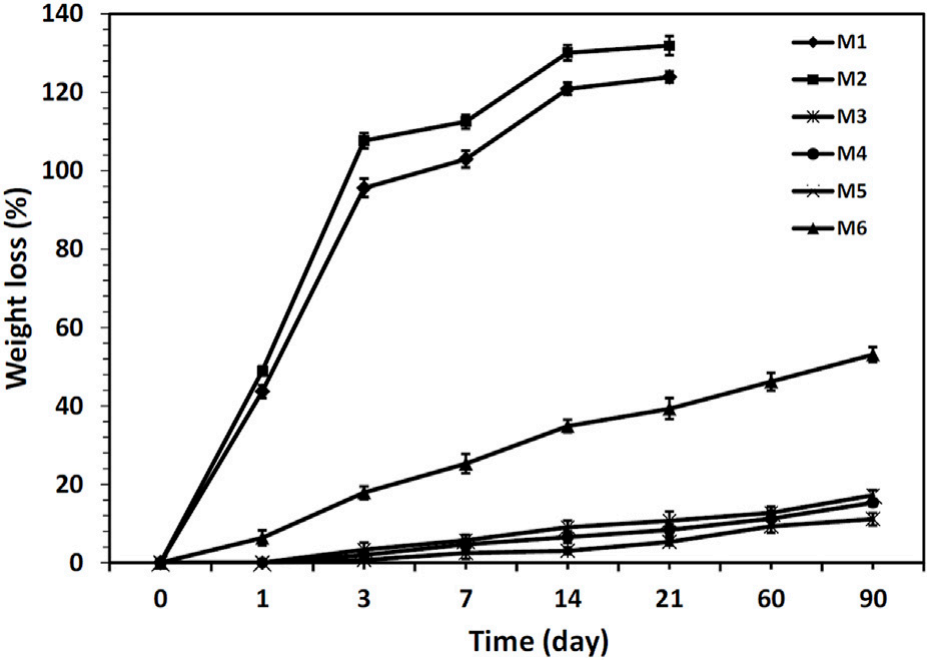
Weight loss of different nanofibrous mats during 90 days’ incubation in PBS.

The mass loss of the different nanofibrous mats during *in vitro* degradation in PBS for 90 days is shown in Figure 5. As can be seen, the M1 and M2 nanofibrous mats were completely degraded after 21 days which can be attributed to the hydrophilic nature of CS and PEO. However, mass losses of M3, M4, and M5 mats were not significant after 90 days. This can be mainly attributed to the physical barrier function of PCL, which effectively prevents water penetration into the mats. (Shoja et al., 2015). Herein, the plasma-treated CS + PEO/PCL nanofibrous mat containing RSV (i.e., sample M6) showed reasonable degradation behavior during the investigated period. These results are in close agreement with the samples’ capacity to absorb water (Figure 4A). An increase in water absorption capacity makes it easier for water to penetrate the nanofibers, which significantly improves the hydrolysis process (Recek et al., 2016).

**FIGURE 6:**
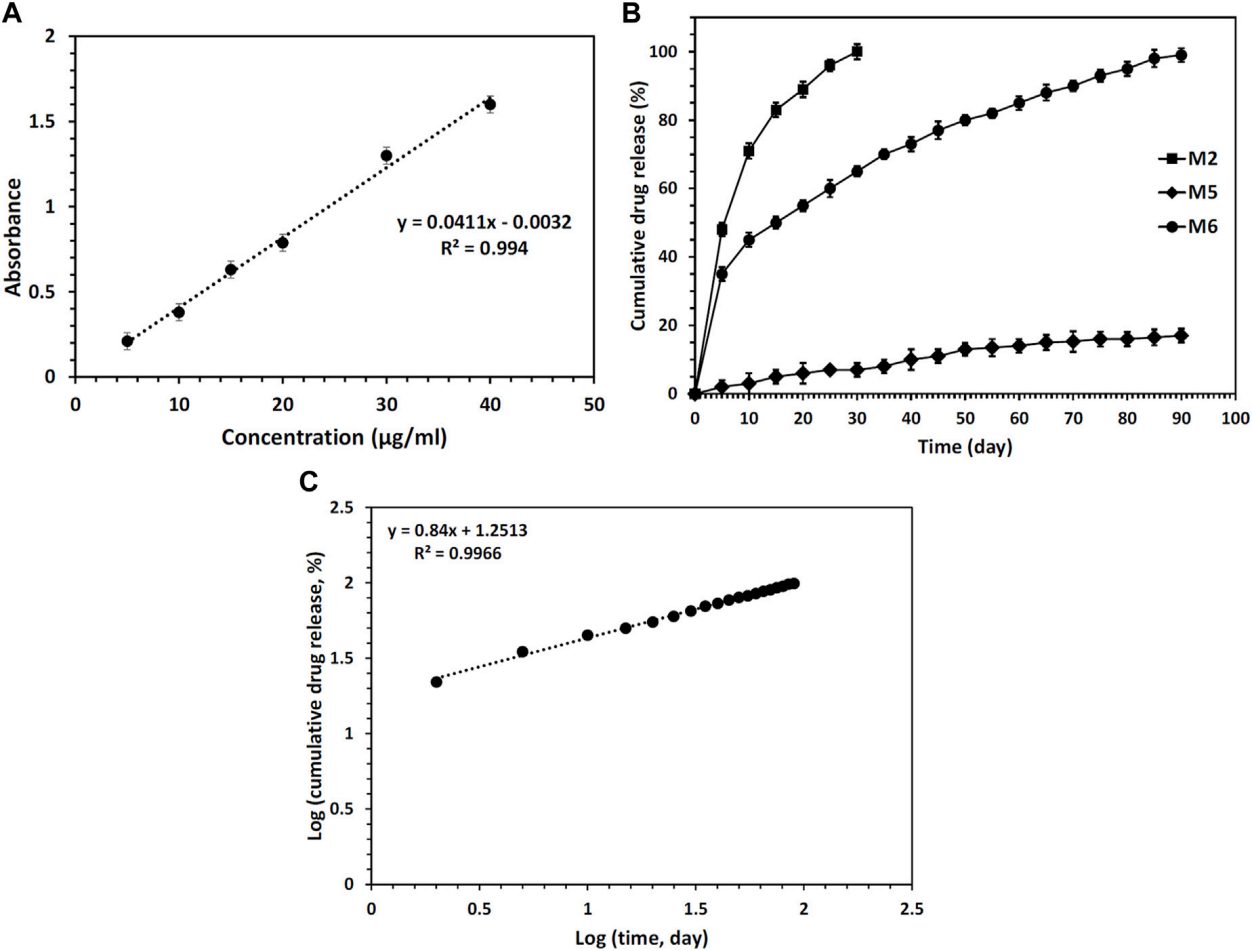
**(A)** Calibration curve of RSV drug, **(B)** RSV release profiles from different nanofibers mats during 90 days of incubation in PBS, and **(C)** analysis of the RSV release data by the Korsmeyer-Peppas model for sample M6.

When aiming to fabricate scaffolds for tissue engineering purposes, a sustained DDS utilizing core/shell electrospun nanofibers holds great promise. This technology has the potential to direct the targeted differentiation of stem cells and facilitate the vascularization of tissue engineering scaffolds, both *in vitro* and *in vivo*. By employing core/shell electrospun nanofibers, it becomes possible to finely design the core/shell structure, enabling precise control over the timing and rate of drug release. This level of control offers significant advantages in tailoring the DDS to meet specific therapeutic requirements. To study the drug release profile, fabricated nanofibrous mats were analyzed. First, the calibration curve of the RSV drug was determined. The obtained results are shown in Figure 6A. Then, the encapsulation efficiency of RSV was determined for different nanofibrous mats containing RSV. Values of about 88, 85, and 87% for samples M2, M5, and M6, respectively, were obtained. Other studies have shown that the encapsulation efficiency was high for nanofibers fabricated via the coaxial electrospinning method due to a high possibility of drug entrapment into the core/shell structure. The samples’ cumulative release profile is presented in Figure 6B. A burst release of RSV was found for RSV-incorporated CS + PEO mats (sample M3) because of the high dissolution rate of CS and PEO in PBS. Herein, about 100% of the drug was released within 30 days. However, unlike CS, PCL does not degrade quickly in PBS, therefore as expected the amount of drug released from the M5 nanofibrous mats was negligible. On the contrary, the plasma-treated CS + PEO/PCL nanofibrous mats containing RSV (sample M6) showed a sustained release profile. The drug was completely released within 90 days. The plasma-treated PCL shell provided a barrier to rapid degradation of the CS + PEO core, whilst the modified PCL enabled water penetration into the core. The data obtained from the kinetics model for RSV release from sample M6 exhibited a release profile that closely followed the Korsmeyer-Peppas model, as indicated by an *R* ^2^ value of 0.9966 (Figure 6C). The value of *n,* determined to be 0.84, suggests a non-Fickian drug release process with the involvement of both diffusion and swelling-controlled release mechanisms (Dash et al., 2010). These findings support the notion that the drug release from the core/shell electrospun nanofibers is a complex and multi-faceted process.

**FIGURE 7:**
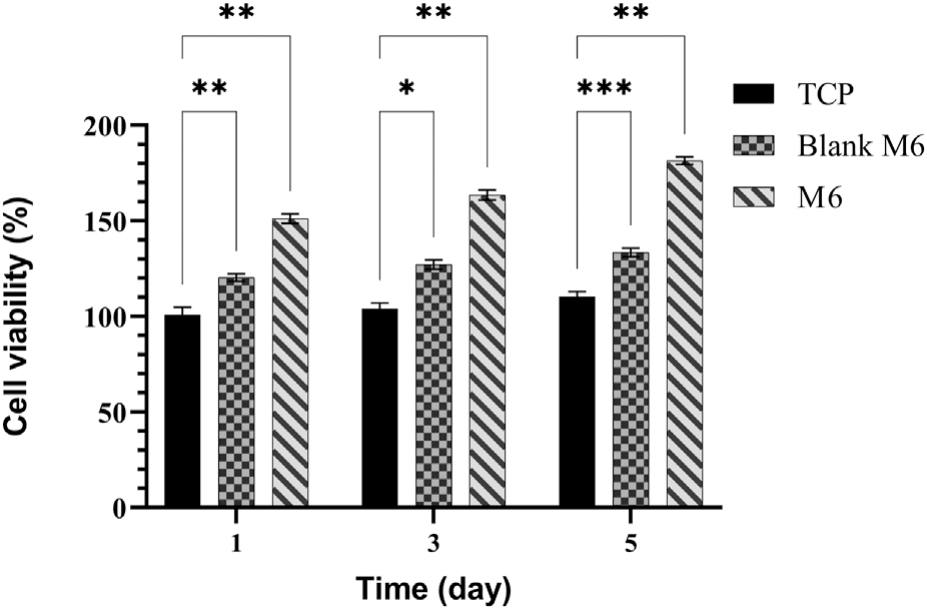
MTT assay for MSCs proliferation on TCPs, Blank M6, and M6 nanofibrous mats. Results are reported as mean ± SD of three independent experiments (*p < 0.05, **p < 0.01, and ***p < 0.001). The value for TCP on 2D cell culture at day 1 is taken as 100% and the other values are presented as a fold increase relative to that.

**FIGURE 8:**
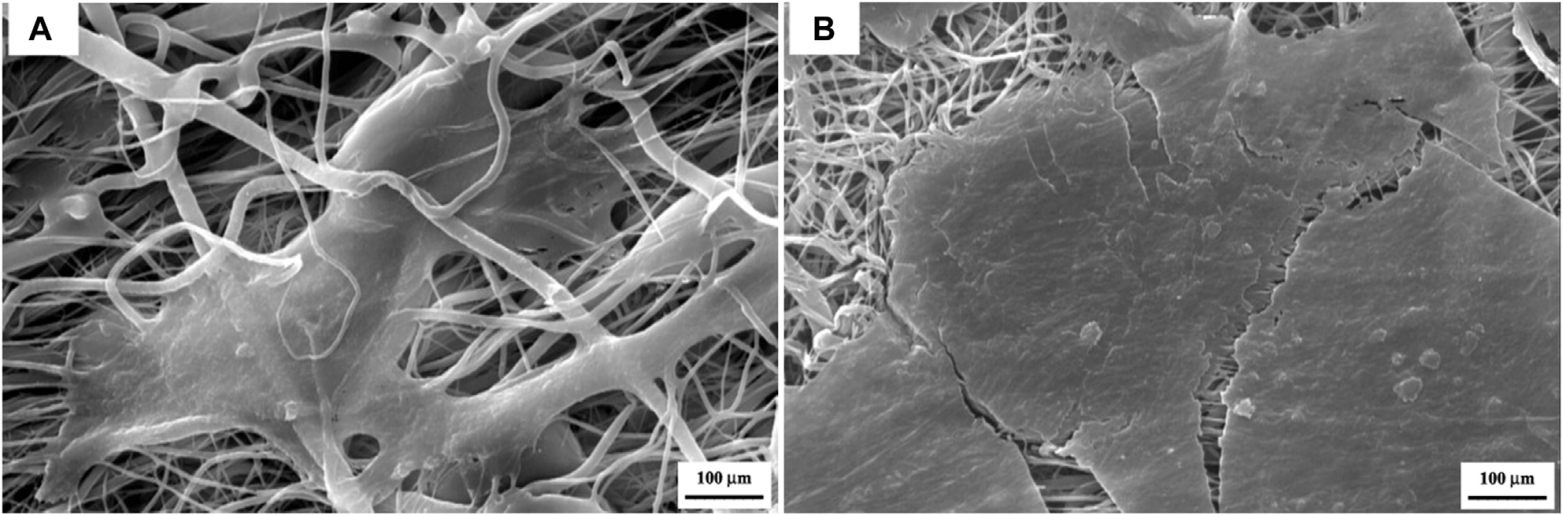
SEM micrographs showing cell attachment on the surface of the CS + PEO/PCL nanofibrous mat containing RSV after 3 days of cell incubation, **(A)** before plasma treatment, and **(B)** after plasma treatment.

According to the release profiles, the plasma-treated CS + PEO + RSV/PCL core/shell nanofibrous mat (sample M6) was considered for additional biological studies, and its cellular activity and osteogenesis were evaluated in comparison with the similar structure not treated with plasma (sample Blank M6). MTT results of MSCs seeded on samples M6 and Blank M6 compared with TCP as the control, for 5 days of culture, are illustrated in Figure 7. In both nanofiber substrates, cell viability was higher than TCP, suggesting that these substrates are nontoxic. The higher surface area and similarities to the ECM structure in nanoscale fibers lead to improved cellular responses (Rezk et al., 2019). Besides, the viability of cells exposed to the plasma-treated CS + PEO + RSV/PCL nanofibrous mat (sample M6) was significantly higher than that of the untreated one (sample Blank M6). According to MTT data, the released RSV was within the range required to encourage cell proliferation and enhance viability without having any negative impacts on MSC behavior. According to previous investigations (Monjo et al., 2010a; Rezazadeh et al., 2019), the effects of RSV on cell proliferation and toxicity are dependent on the dosage. RSV has been found to promote cell proliferation in MC3T3-E1 cells at lower doses, whereas higher concentrations can lead to cytotoxicity. The cytotoxic effect of concentrated statins, including RSV, can be attributed to a substantial reduction in cholesterol levels, which is a crucial component for the maintenance of cell membranes (Monjo et al., 2010a). In addition, Figure 8 shows the morphologies of MSCs after 3 days of culture on the surface of samples Blank M6 and M6. As desired, cells adhered effectively to both substances, with greater propagation occurring on the plasma-treated sample’s surface (M6, Figure 8B).

**FIGURE 9:**
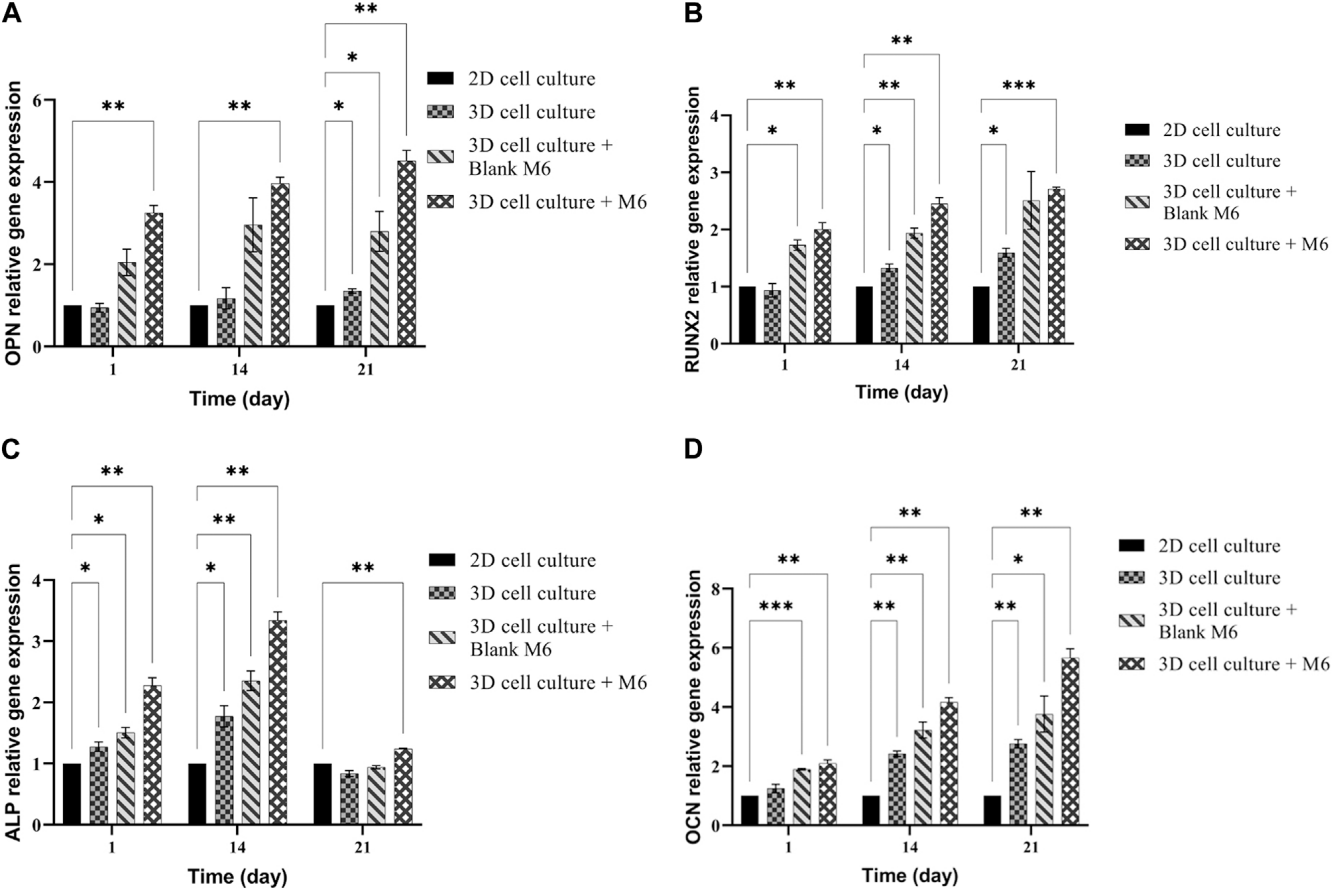
mRNA expression analysis by qPCR of **(A)** OPN, **(B)** RUNX2, **(C)** ALP, and **(D)** OCN normalized for GAPDH on 2D cell culture, 3D cell culture with or without M6. Results are reported as a fold increase relative to 2D cell culture (mean ± SD, *n* = 3). *p < 0.05, **p < 0.01, ***p < 0.001.

Osteogenic differentiation of MSCs on the plasma-treated CS + PEO + RSV/PCL core/shell nanofibrous mat (M6) was quantitatively analyzed by measuring levels of RUNX2, osteopontin (OPN), alkaline phosphatase (ALP), and osteocalcin (OCN) with RT-PCR. The obtained results (Figure 9) were compared with those for plasma-treated CS + PEO/PCL core/shell nanofibrous mat (Blank M6). On day 21, the expression of OPN as a late marker of osteogenic differentiation, reached its peak level (Figure 9A). The peak in OPN expression for MSCs cultivated on RSV-loaded mats, indicates the advanced stage of osteogenic differentiation and the maturation of the cultured cells towards bone-forming cells. This is in agreement with previous studies showing that OPN is significantly upregulated during the osteogenic differentiation process (Kang et al., 2015; Shalumon et al., 2015). RUNX2 is a marker for osteogenic differentiation that appears early on and upregulates osteocalcin, a key component of the bone ECM and ALP. As seen in Figure 9B, RUNX2 expression peaked in the RSV-loaded mat on day 21, relative to non-loaded nanofibers. However, there was no significant difference between days 7, 14, and 21. This finding proposes that RSV may have a role in encouraging stronger osteogenesis at an early stage. Earlier studies have drawn comparable findings, indicating that RSV along with other statins might enhance osteogenesis by elevating the level of RUNX2 expression which is a crucial factor linked with the development of osteoblasts. (Shalumon et al., 2015; Kalani et al., 2019). ALP is commonly regarded as an early/intermediate sign of osteoclastogenesis and is crucial for the mineralization of the substrate. As seen in Figure 9C, the RSV-containing nanofibers displayed a high expression of ALP (at day 14) that was statistically different from the mat without RSV. As anticipated, throughout the incubation time there was no noticeable difference between the ALP expressions of the sample without RSV. Over 21 days, OCN as a late marker of osteogenesis showed a rising tendency in both formulations, but the sample containing RSV considerably upregulated OCN expression relative to the one without RSV (Figure 9D). According to these findings, the RSV-containing sample shows increased osteocalcin gene expression (Shalumon et al., 2015; Kalani et al., 2019).

**FIGURE 10:**
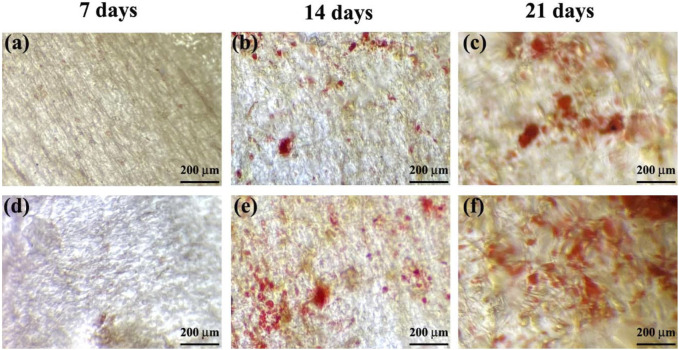
Alizarin red staining of MSCs on, **(A–C)** Blank M6, and **(D–F)** M6 nanofibrous mats after 7, 14, and 21 days of incubation.

**FIGURE 11:**
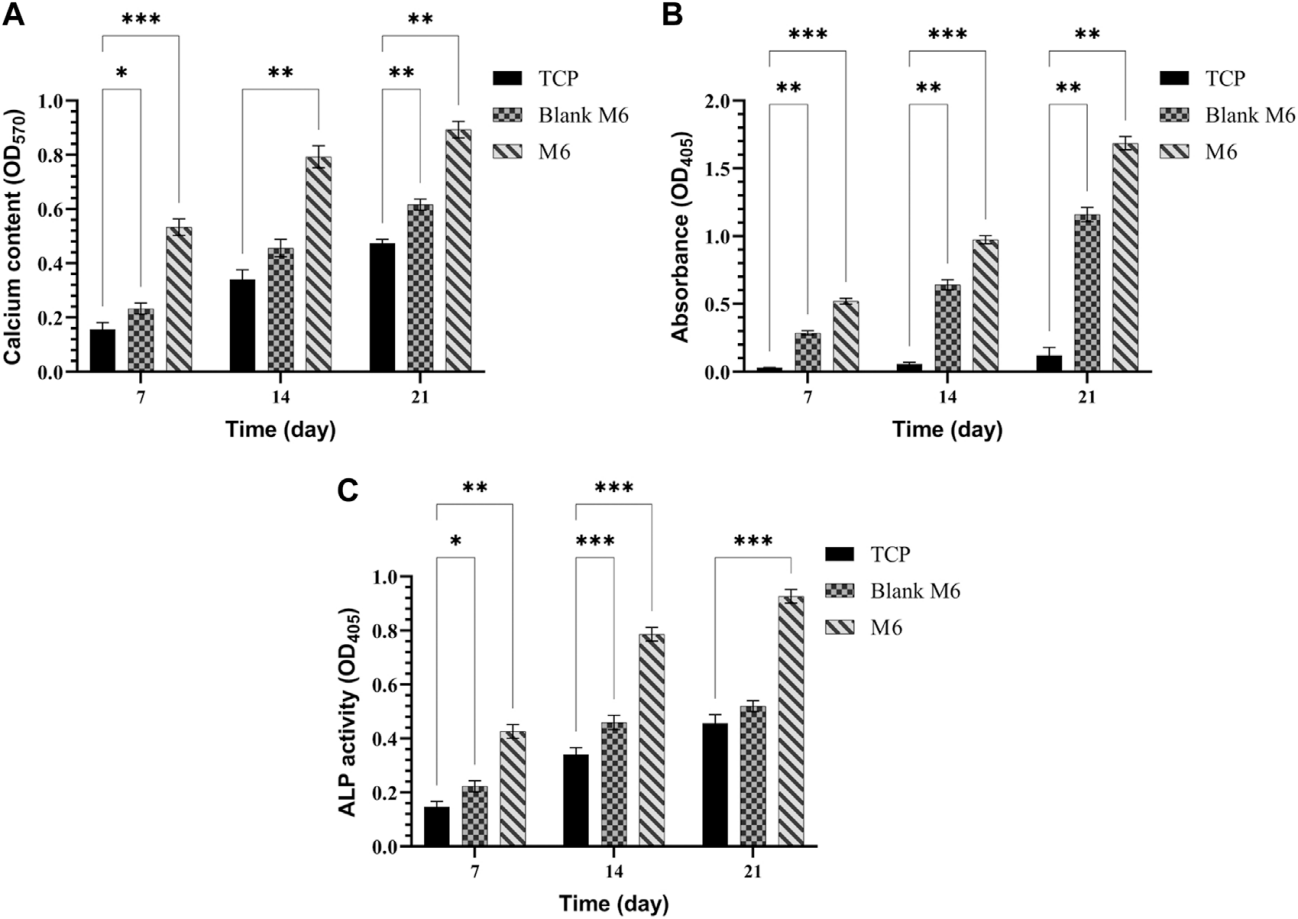
**(A)** Calcium content, **(B)** quantitative Alizarin red staining, and **(C)** ALP activity produced by MSCs cultured on TCPs, Blank M6, and M6 nanofibrous mats after 7, 14, and 21 days. Results are reported as mean ± SD of three independent experiments (*p < 0.05, **p < 0.01, and ***p < 0.001). 2D cell culture was used for all the experiments.

In the examined samples on days 7, 14, and 21 of culture, the appearance of mineralized ECM, which serves as a late marker of osteogenic differentiation of MSCs, was observed (Figure 10). Notably, in all incubation durations, the production of ARS-calcium complex (indicated by red/purple dots) was higher in the M6 nanofibrous mat compared to the Blank M6. Moreover, the presence of calcium deposits (Figure 11A), quantitative Alizarin red staining (Figure 11B), and ALP activity (Figure 11C) by MSCs were significantly higher on day 21 in sample M6. These findings strongly support the potential role of RSV in enhancing osteogenic differentiation, as indicated by the increased mineralization and ALP activity in the M6 nanofibrous mat.

## 4 Conclusion

This study focused on the fabrication of plasma-treated nanofibers using the coaxial electrospinning process, resulting in core/shell structures. The nanofibers exhibited a smooth and uniform morphology without any visible beads, with a core diameter of approximately 370 nm and a shell thickness of around 70 nm. TGA and FTIR analyses confirmed the presence of different components within the fabricated nanofibers. The drug release profile demonstrated that the incorporation of RSV in the core layer of the nanofibers led to a sustained release pattern with a reduced initial release. The use of these nanofibers as a delivery system for RSV showed enhanced proliferation of MSCs and improved osteogenic differentiation, as evidenced by the upregulation of OPN, OCN, ALP, and RUNX2 gene expression, as well as increased ARS staining. Based on the findings, it is proposed that the CS + PEO + RSV/PCL core/shell nanofibrous mat can serve as an effective carrier for the sustained release of RSV, making it suitable for applications in bone tissue engineering such as guided bone regeneration, bone fracture healing, and localized drug delivery to the damaged bone site.

## Data Availability

The raw data supporting the conclusions of this article will be made available by the authors, without undue reservation.
